# Postpartum physical intimate partner violence among women in rural Zambia

**DOI:** 10.1002/ijgo.12654

**Published:** 2018-09-05

**Authors:** Michelle L. Munro‐Kramer, Nancy Scott, Carol J. Boyd, Philip T. Veliz, Sarah M. Murray, Gertrude Musonda, Jody R. Lori

**Affiliations:** ^1^ Department of Health Behavior and Biological Sciences University of Michigan School of Nursing Ann Arbor MI USA; ^2^ Department of Global Health Boston University School of Public Health Boston MA USA; ^3^ Applied Biostatistics Laboratory University of Michigan School of Nursing Ann Arbor MI USA; ^4^ Department of Mental Health Johns Hopkins Bloomberg School of Public Health Baltimore MD USA; ^5^ Africare Zambia Lusaka Zambia

**Keywords:** Intimate partner violence, Maternity waiting homes, Mental health, Physical violence, Postpartum depression, Zambia

## Abstract

**Objective:**

To examine the demographic characteristics and mental health of women in rural Zambia who experienced physical intimate partner violence (IPV) postpartum.

**Methods:**

The present secondary analysis was conducted using baseline data from an impact evaluation of a maternity waiting home intervention in rural Zambia. A quantitative household survey was conducted over 6 weeks, from mid‐April to late May, 2016, at 40 rural health facility catchment areas among 2381 postpartum women (13 months after delivery; age ≥15 years).

**Results:**

A total of 192 (8.1%) women reported experiencing any type of physical IPV in the preceding 2 weeks; 126 had experienced severe physical IPV (had been kicked, dragged, beat, and/or choked by a husband or partner). High levels of depression were recorded for 174 (7.3%) women in the preceding 2 weeks. Being a female head of household was associated with an increased likelihood of experiencing severe physical IPV (aOR 2.64, 95% CI 1.70–4.10). Women with high depression scores were also at an increased risk of experiencing any physical IPV (aOR 17.1, 95% CI 8.44–34.9) and severe physical IPV (aOR 15.4, 95% CI 5.17–45.9).

**Conclusion:**

Future work should consider the implications of government and educational policies that could impact the screening and treatment of pregnant women affected by all forms of physical IPV and depression in rural Zambia.

## Abstract 2

Postpartum physical intimate partner violence among women in rural Zambia was associated with being a female head of household and high levels of depression.

## 1 INTRODUCTION

Intimate partner violence (IPV)—defined as physical, sexual, psychological, or economic abuse perpetrated by a current or former partner—is the most common form of violence against women globally, with approximately one in three women experiencing physical IPV, sexual IPV, or non‐partner sexual violence in their lifetime.^1^ Such behavior is not only a violation of human rights but also a public health challenge that has both acute and chronic implications for reproductive, physical, and mental health. Previous studies have demonstrated associations between IPV and gynecologic disorders, adverse pregnancy outcomes, chronic pain, and mental health complications.^2^, ^3^ Further, mounting evidence suggests that trauma experienced across the lifespan, including that caused by IPV, contributes to chronic disease and premature ageing, which in turn can lead to increased morbidity and premature death.^4^, ^5^

The prevalence of IPV during pregnancy is 2%–57% across African countries, with a meta‐analysis yielding an overall prevalence of 15.23%.^6^ Experiencing IPV during pregnancy and in the immediate postpartum period is of particular concern given the adverse health effects for both mother and child. The intergenerational effects of IPV are also well established; IPV during the perinatal period has been linked to negative developmental and health effects among offspring,^7^ including future episodes of mental health problems.^8^ Witnessing IPV can also affect the attitudes and behaviors of children, increasing the likelihood that they will either perpetrate or experience IPV as adults.^9^

Further complicating the issue is the bidirectional association between IPV and mental health. Depression has been linked to IPV both as a consequence of experiencing such behavior and as a risk factor.^10^ The published literature on IPV in Sub‐Saharan Africa has primarily focused on patriarchal gender norms and demographic factors as potential predictors of IPV and has not explored the role of mental health.^11^ However, a South African study^12^ found that high levels of depressive symptoms were associated with an increased risk of experiencing IPV among postpartum women.

In Zambia, 47% of women and 32% of men agreed that a husband is justified in beating his wife for at least one specified reason.^13^ In addition, the overall prevalence of IPV reported among women aged 15–49 years who were currently or previously married was 47%, with 10% of these women experiencing IPV during pregnancy.^13^

Previous research on IPV in Zambia has mainly sought to understand the sociodemographic factors associated with such behavior. The Zambian Demographic and Health Survey of 2013–2014^13^ found that women with a large number of children and low levels of education were more likely to experience sexual and physical IPV than their peers with more education and a smaller number of children. An analysis of the Zambian Demographic and Health Survey data from 2001 to 2002^14^ found the following factors to be associated with experiencing physical IPV in the past year: age 15–19 years or 45–49 years; urban dwelling; low level of education; women with tolerant attitudes toward physical IPV; and women with low level of autonomy (e.g. on health issues).

Despite the frequency of IPV during the perinatal period, only limited investigation has been conducted among reproductive‐aged women living in rural areas of Sub‐Saharan Africa, including Zambia. In addition, little evidence exists regarding the prevalence of IPV and its relationship with depression among postpartum women in Zambia, particularly among those living in remote areas.

An understanding of the driving forces behind IPV in Sub‐Saharan Africa is imperative to meet international benchmarks, including the United Nations Sustainable Development Goal 5 (gender equality).^15^ Given that the perinatal period is critical for maternal and child health, we evaluated the demographic and mental health risk factors associated with IPV in three districts of Zambia (Eastern, Southern, Luapula) currently receiving increased programmatic infrastructure surrounding maternal health.

The aims of the present study were to determine the prevalence of postpartum physical IPV among women in rural Zambia and to evaluate demographic characteristics and mental health status (e.g. depression) within this population.

## 2 MATERIALS AND METHODS

The present secondary analysis included baseline data from an impact evaluation of a maternity waiting home (MWH) intervention in rural Zambia.^16^ A cross‐sectional quantitative household survey was conducted over a 6 week period between mid‐April and late May, 2016, at 40 rural health facility catchment areas before the establishment of a maternity waiting home intervention designed from formative research in seven Saving Mothers, Giving Life districts in Zambia.^17^, ^18^ Saving Mothers, Giving Life is a country‐wide initiative to improve maternal morbidity and mortality. Maternity waiting homes are physical structures built near rural health facilities that provide women with a place to stay before and after delivery. The districts targeted in the present study were Choma, Kalomo, Lundazi, Mansa, Nyimba, Pemba, and Chembe. Ethical approval for the household survey was obtained before data collection from the institutional review boards of the University of Michigan (Ann Arbor, USA) and Boston University (Boston, USA), as well as the research ethics committee of ERES Converge, Lusaka, Zambia. Informed consent was obtained from all participants before data collection.

A multistage random sampling approach was used to select a representative sample of women living in remote dwellings within the 40 health facility catchment areas. First, all villages were geocoded to identify those located at least 9.5 km (rounded up to nearest kilometer) from the catchment area health facility by the most direct travel routes using ArcGIS Online (Esri, Redlands, CA, USA). Villages were then randomly selected from this sample using probability proportionate to population size. A maximum of 10 clusters was selected per each health facility catchment area. Second, all eligible households within the selected villages were listed with the assistance of village leadership and community members. Systematic random sampling was then used to select every nth household from the list to approach for participation until the required sample size for that village was reached—full details of the process have been published previously.^16^

Eligible women were from unique households, had delivered within the preceding year, and were aged 15 years or older. Participants were excluded if they were unwilling or unable to provide informed consent. Although the survey asked about deliveries within the past 12 months, birthdates are often approximated in Zambia; therefore, the range of the present sample went up to 13 months. Households were defined as usually cooking together based on their cultural background. If there was more than one eligible woman in the household, one of them was randomly selected for inclusion in the present study.

A team of Zambian research assistants, literate in both the appropriate local languages (Bemba, Nyanja, Tonga and Tumbuka) and English, were trained in human participant protection and data collection methods during a 5‐day training program. Data were captured electronically using SurveyCTO Collect version 2.212 (Dobility, Cambridge, MA, USA), which was installed on encrypted tablet devices.

Participants were initially contacted through personal visits from the research assistants. They were then invited to select a space where they felt comfortable and could speak in private. All survey questions were read aloud to the participants by the research assistants; each survey took approximately 45 minutes to complete. Participants received a small token of appreciation, valued at approximately US $2, for their time.

Household and individual sociodemographic variables assessed were household size, marital status (married or cohabitating, divorced, separated, widowed, and never married), number of deliveries, number of wives shared with a husband, and age. These variables were selected on the basis of past research showing them to be predictors of physical IPV in Sub‐Saharan Africa.^13^, ^19^ Head of household was ascertained by the question “Are you the head of household?” Women who responded “no” were then asked, “What is your relationship to the head of household?”

The scale used to assess depression comprised four items asking how often women felt lonely, cried, or experienced a lack of interest in activities in the preceding 2 weeks. These items were adapted from the Hopkins Symptom Checklist^20^ and the Center for Epidemiological Studies–Depression Scale,^21^ both of which are commonly used to measure depression in Sub‐Saharan Africa. Women responded using a four‐point scale: 0 (never), 1 (once in a while), 2 (more than half the time), and 3 (almost always). A scale total was constructed using the average of these four items: 0 (no depression), 0.01–0.75 (low levels of depression), 0.76–1.00 (moderate levels of depression), and 1.01–3.00 (high levels of depression), with a Cronbach α of 0.819.

Data on recent physical IPV were collected by asking how often women had been pushed, shoved, or slapped by their husband or partner in the previous 2 weeks. They were also asked how often they had been kicked, dragged, beaten, or choked by their husband or partner in the previous 2 weeks (classified as severe physical IPV). Participants could select from four categories that ranged from “never” to “almost always.”

The data were analyzed using Stata version 14.0 (StataCorp, College Station, TX, USA). Descriptive statistics and adjusted odds ratios (aORs) were calculated to examine the associations between sociodemographic characteristics, depression, and physical IPV (none vs at least one incident). List‐wise deletion was used to account for the small amount of missing data. Binary logistic regression models were used to estimate aORs and 95% confidence intervals (CIs) while controlling for the sociodemographic characteristics. The Stata robust cluster estimator was used to account for clustering within each of the seven districts. *P*<0.05 was considered statistically significant.

## 3 RESULTS

The response rate among the 2741 women invited to participate in the present study was 86.9%, giving a final sample size of 2381. Of the women who were eligible to participate but who did not respond, 280 (10.2%) were unavailable owing to their work in the fields during harvest, 60 (2.2%) refused participation, and 20 (0.7%) withdrew after beginning the survey or else had incomplete surveys and so were dropped from the analysis.

Table 1 outlines the characteristics of the 2381 participants, whose age range was 15–49 years (mean 26.1 ± 7.0 years). **[**The majority of the women were married (2092 [87.9%]) and had undergone four or more deliveries (1068 [44.9%]). Households were primarily led by a male (1824 [76.6%]) and comprised at least seven people (1166 [49.0%]). Overall, 192 (8.1%) participants reported experiencing any physical IPV and 126 (5.3%) reported experiencing severe IPV over the past 2 weeks. A total of 174 (7.3%) women reported high levels of depression.

**Table 1:** Characteristics of the study population (n=2381).^a^

Characteristic	Value	95% confidence interval
Physical IPV^b^		
Any	192 (8.1)	5.6–11.6
Severe	126 (5.3)	3.4–8.1
Depression		
None	1231 (51.7)	45.7–58.4
Low	820 (34.4)	32.3–37.2
Moderate	135 (5.7)	4.2–7.8
High	174 (7.3)	4.8–10.9
Missing	21	
Mean number of individuals in the household	6.99	5.83–8.15
Marital status		
Married	2092 (87.9)	82.1–92.1
Not Married	284 (11.9)	7.8–17.8
Missing	5	
Mean number of deliveries	3.59	3.24–3.94
Head of the household		
Male	1824 (76.6)	72.5–80.3
Female	232 (9.7)	5.6–16.2
Unknown	324 (13.6)	9.4–19.4
Missing	1	
Age, y	26.1	25.5–26.7
Level of education		
None	362 (15.2)	10.1–22.2
Some primary	968 (40.7)	29.1–53.5
At least completed primary	1044 (43.8)	28.3–60.8
Missing	7	

Abbreviation: IPV, intimate partner violence.

a Values are given as number (percentage) or mean, unless indicated otherwise.

b Any type of physical IPV was defined as pushed, shoved, and/or slapped by a husband or partner in the past 2 wk; severe physical IPV was defined as kicked, dragged, beat, and/or choked by a husband or partner in the past 2 wk.

The binary logistic regression is shown in Table 2. Data on variables of interest were missing for 38 of the 2381 women; therefore, these participants were excluded from the analysis. Modeling indicated that being a female head of household was associated with an increased likelihood of experiencing any physical IPV (OR 1.86, 95% CI 1.16–2.99) and severe physical IPV (aOR 2.64, 95% CI 1.70–4.10). Further, there was a strong link between depression and physical IPV, with the odds of experiencing such behavior increasing as the depression score increased. For participants with high levels of depression, the aORs were 17.1 (95% CI 8.44–34.9) for any physical IPV and 15.4 (95% CI 5.17–45.9) for severe physical IPV.

**Table 2:** Correlates of physical intimate partner violence (n=2343)

Variable	Any physical IPV^a^		Severe physical IPV^b^	
	OR (95% CI)^c^	aOR (95% CI)^d^	OR (95% CI)^c^	aOR (95% CI)^d^
Depression				
None	1.00	1.00	1.00	1.00
Low	3.33 (1.50–7.38)^e^	3.37 (1.51–7.49)^e^	2.87 (0.87–9.48)	2.97 (0.93–9.49)
Moderate	13.7 (5.68–33.2)^f^	14.4 (5.89–35.4)^f^	14.5 (3.95–53.3)^f^	15.6 (4.47–54.6)^f^
High	16.9 (8.50–33.9)^f^	17.1 (8.44–34.9)^f^	15.6 (5.40–45.4)^f^	15.4 (5.17–45.9)^f^
Mean number of individuals in the household	1.00 (0.95–1.05)	1.00 (0.96–1.05)	0.99 (0.94–1.03)	0.97 (0.93–1.02)
Marital status				
Married	1.00	1.00	1.00	1.00
Not married	1.74 (0.98–3.09)	1.46 (0.57–3.78)	2.72 (1.30–5.70)^e^	1.42 (0.50–4.03)
Mean number of deliveries	0.95 (0.88–1.02)	0.93 (0.83–1.04)	0.90 (0.78–1.04)	0.91 (0.77–1.09)
Head of the household				
Male	1.00	1.00	1.00	1.00
Female	1.86 (1.16–2.99)^e^	1.42 (0.79–2.56)	3.31 (1.95–5.61)^f^	2.64 (1.70–4.10)^f^
Unknown	1.35 (0.73–2.51)	0.72 (0.43–1.19)	2.32 (1.07–5.04)^g^	1.39 (0.55–3.50)
Mean age, y	0.99 (0.96–1.02)	1.00 (0.95–1.04)	0.98 (0.92–1.03)	1.0 (0.93–1.07)
Education				
None	1.00	1.00	1.00	1.00
Some primary	0.86 (0.55–1.34)	0.80 (0.477–1.34)	0.67 (0.40–1.11)	0.59 (0.31–1.12)
At least completed primary	1.15 (0.80–1.67)	1.04 (0.734–1.47)	1.10 (0.79–1.53)	0.92 (0.75–1.12)

Abbreviations: aOR, adjusted odds ratio; CI, confidence interval; IPV, intimate partner violence; OR, odds ratio.

a Defined as pushed, shoved, and/or slapped by a husband or partner in the past 2 wk.

b Defined as kicked, dragged, beat, and/or choked by a husband or partner in the past 2 wk.

c Binary logistic regression models were used to calculate ORs.

d Binary logistic regression models for aORs included the following variables: depression, mean household size, marital status, mean number of deliveries, head of household, mean age, and highest level of education.

e *P*<0.01.

f *P*<0.001.

g *P*<0.05.

## 4 DISCUSSION

The present study found prevalence rates for any physical IPV and severe physical IPV of 8.1% and 5.3%, respectively, among a group of postpartum women living in rural Zambia. The risk of experiencing such behavior was increased by being a female head of household or high levels of depression.

Most IPV prevalence studies have used the past 12 months to indicate “recent” experiences of such behavior.^12^, ^22^ By contrast, the present study examined IPV during the past 2 weeks. Despite this marked difference in time frame, the current prevalence rates were comparable to previous reports of past year physical and/or sexual IPV in the postpartum period (5.2%–10.5%).^12^, ^22^

The present study found that postpartum women in female‐headed households were more likely to report physical IPV, specifically severe physical IPV, in the preceding 2 weeks than those in male‐headed households. This observation was in agreement with a study conducted in Haiti, which found that women in communities with a high proportion of female‐headed households showed increased risk of sexual IPV.^23^ Likewise, communities in the USA with large proportions of female‐headed households tend to have increased levels of violence against women.^24^ Most of the hypotheses for this association have centered on poverty and the financial status of communities with a high proportion of female‐headed households. However, as less between‐community variation in financial status exists in rural Zambia, the relationship between IPV and head of household has been measured at an individual level rather than the population level.^13^ Nontraditional gender roles might also influence the association between head of household and IPV. Previous work in Zambia found that the most common reasons given by reproductive‐aged women for justifying IPV were when women had transgressed from their expected gender roles; for example, equal autonomy related to household decisions.^25^

The present study also found an association between physical IPV and depression, although a causative link could not be established owing to the cross‐sectional nature of the data. Postpartum depression can have a lasting impact on individuals, affecting their ability to work, care for their family, and contribute to society. For female caregivers, postpartum depression can have negative effects on parenting and safety practices (e.g., using an infant car seat, childproofing the home),^26^ as well as on the cognitive development of their offspring.^27^ Depression is one of the leading causes of disability and is a frequent occurrence among women during the postpartum period.^26^ In many low‐resource countries in Africa, including Zambia, mental health problems are stigmatized and not prioritized by the healthcare system.^28^ In rural regions of Zambia, clinics are often staffed by midwives without formal training in mental health who are overburdened providing perinatal care. Further, Zambia has placed very little emphasis on mental health issues. Despite having a mental health policy in place since 2005, data collected in 2011 indicated that Zambia had only 0.025 psychiatrists per 100 000 individuals and just 0.38% of the government health expenditure was spent on mental health.^28^

Current policies and laws related to IPV in low‐income countries have shown limited success in changing conventional gender norms and attitudes. For example, Zambia implemented the Anti‐Gender‐Based Violence Act in 2011 and the National Gender Policy in 2014; however, there has been little change in the rates of IPV.^13^ The results of the present study indicated a need to focus on both IPV and mental health among Zambian women of reproductive age. In addition, screening for IPV and depression by relevant healthcare providers (e.g. midwives) during the perinatal period is required.

Interpretation of the present study findings was constrained by some limitations. The cross‐sectional design provided only a snapshot of the experiences of physical IPV among postpartum women in rural Zambia, although it could be argued that the findings still made an important contribution to understanding their lives. As a secondary analysis, the present study was limited by the variables in the database, with only two questions posed regarding physical IPV. As a result, the present study was unable to capture all forms of IPV, including sexual, psychological, and economic abuse. Depression was measured using an adapted four‐item scale to minimize respondent burden in the original impact evaluation study. Although these items were drawn from scales validated in Zambia, by selecting only a subset of items, other important aspects of depression could have been missed.

Nonetheless, these limitations should be weighed against the strengths of the present study. To our knowledge, the present study was the first to demonstrate the experiences of postpartum physical IPV among women in rural Zambia and the potential association between non‐traditional gender roles and physical IPV in this country. The present study also demonstrated a strong link between depression and physical IPV. Finally, the current findings highlighted a need for policy changes to impact the lives of women and their families in Zambia.

In conclusion, the results of the present study should help to inform future programmatic and intervention efforts aimed at changing individual, community, and societal factors associated with IPV. Future work should consider the implications of governmental and educational policies that could influence the screening and treatment of women affected by all forms of IPV in rural Zambia. In addition, prevalence studies should incorporate a longitudinal timeline covering the entire perinatal period. Finally, it will be important to consider suitable venues to educate women about IPV and postpartum depression.

## AUTHOR CONTRIBUTIONS

MLM‐K contributed to the design of the study, data analysis and interpretation, and writing the manuscript. NS contributed to designing the study, data collection and interpretation, and writing the manuscript. CJB, SMM, and JRL contributed to designing the study, the interpretation of data, and writing the manuscript. PTV contributed to the analysis and interpretation of data, and writing the manuscript. GM contributed to designing the study, data collection, and writing the manuscript. All authors provided final approval of the version to be published and agreed to be accountable for the accuracy and integrity of the work.

## CONFLICTS OF INTEREST

The authors have no conflicts of interest.
